# New Insights into Metabolic Alterations and Mitochondria Re-Arrangements in Pancreatic Adenocarcinoma

**DOI:** 10.3390/cancers15153906

**Published:** 2023-08-01

**Authors:** Ilaria Dando, Elisa Dalla Pozza

**Affiliations:** Department of Neuroscience, Biomedicine and Movement Sciences, University of Verona, 37134 Verona, Italy

Among the most aggressive cancer types, pancreatic ductal adenocarcinoma (PDAC) represents one with the highest lethality due to its resistance to therapies and to the frequent metastatic spread. Despite many efforts having been applied to discover new diagnostic tools and therapeutic strategies, in recent decades, the 5-year survival rate has only been improved from about 5% to 10% [1]. In this context, emerging evidence has highlighted the crucial role of metabolic alterations and mitochondria arrangements in cancer evolution, with PDAC representing a model for these discoveries. Indeed, it has been shown that PDAC often exhibits alterations of many metabolic pathways compared to normal cells of the same tissue, including glycolysis, oxidative phosphorylation, amino acids, and lipid metabolism, with the aim of adapting energetic requirements to a new microenvironment for the sustainment of uncontrolled proliferation [2]. The articles published in this Special Issue further encourage the clinical, cellular, and molecular comprehension of the fact that mitochondria, which represent the core of energy production within the cell, are generally altered in cancer, correlating with the stimulation of proliferative signals. Indeed, the review published by Carmona-Carmona et al. was centrally focused on the alterations of mitochondria arrangements, called mitochondria dynamics, in PDAC [2]. Indeed, it has been extensively demonstrated that mitochondria are dynamic organelles that can fuse with each other or can divide into smaller ones based on the specific signal cascades that are, at the same time, regulated upstream by nutrient requirements or intra-/extra-cellular inputs; this phenomenon is amplified and dysregulated in cancer. In this context, PDAC is not an exception since the authors reported that, despite different articles showing opposite results, it appears more evident that cancer cells preferentially support mitochondria fragmentation to sustain tumor needs. Interestingly, in another article, it has been demonstrated that a subpopulation of highly aggressive cancer cells, called cancer stem cells (CSCs) [3], present an even more altered pattern of mitochondria arrangement, by preferring elongated structures with a key role of the fusion protein Opa1, suggesting it as a novel potential therapy [4]. According to the important role of mitochondria in the sustainment of PDAC proliferation, another paper published by Pidinharayil et al. further emphasized that targeting mitochondrial metabolism can have an amplified effect on several metabolic pathways that are crucial for cancer and which can modulate different mechanisms, such as cellular dynamics, bioenergetics, immune education, and retrograde signaling [5]. A different revision study reported that mitochondrial transporters are also important due to their role in the uptake of metabolites and the subsequent alteration of the corresponding metabolic pathways [6]. The authors mainly focused their attention on mitochondrial solute carriers, highlighting their role in metabolic compartmentalization and, hence, in metabolic rewiring. Indeed, they reported a correlation of five mitochondrial transporters and metabolic reprogramming in PDAC, stressing that their targeting could represent an attractive strategy to fight cancer. Specifically, they described the mitochondrial pyruvate carrier (MPC), the glutamine carrier (SLC1A5_Var), the glutamate carrier (GC), the aspartate/glutamate carrier (AGC), and the uncoupling protein 2 (UCP) and their influence on PDAC cell growth and progression. Interestingly, MPC expression is downregulated in PDAC, whereas the expression of the other four transporters is generally upregulated, with evidence that the loss of one or more of them leads to the detriment of PDAC cell growth and proliferation [6]. Additionally, another study presented in this Special Issue shows that the transcription factor EB (TFEB), which is known to act as a master regulator of lysosomal function and autophagy, is also a nutrient sensor, supporting the responses to cellular stress and immune stimuli. Indeed, based on the data showing that TFEB is overexpressed in PDAC cells compared to normal tissue samples, the authors demonstrated that its genetic inhibition resulted in a significant decrease in both glutamine and mitochondrial metabolism, suppressing PDAC growth in vitro and in vivo [7]. This study added a piece of knowledge to the identification of novel therapeutic approaches based on metabolic targets and, more specifically, on glutaminase-mediated glutamine metabolism, which may represent a key target. Another study that identified metabolic proteins as targets for PDAC growth impairment is that by Pacchiana et al., where they tested the in vitro and in vivo effects of novel inhibitors, 3-bromo-isoxazoline derivatives, of the glycolytic enzyme glyceraldehyde-3-phosphate dehydrogenase (GAPDH) [8]. Their results demonstrated that these compounds, in particular AXP-3019, present anti-proliferative effects on PDAC cells and CSCs, without affecting normal fibroblasts. Finally, two other articles presented new insights into the exploitation of metabolic and other pathways, for instance genetic and inflammatory, for cancer treatment by studying two diseases that strictly correlate with PDAC: hepatopancreatobiliary cancer and intraductal papillary mucinous neoplasms (IPMN). Specifically, regarding hepatopancreatobiliary cancer, the authors started from the evidence that in these cancer cells there is a general increase in lipid synthesis and alterations in lipid metabolism associated with lipid droplets’ accumulation. Lipid droplets are intracellular structures that store neutral lipids and acts as molecular messengers and signaling factors, thus mediating proliferation, invasion, metastasis, and chemotherapy resistance. The authors discerned the role of different lipid droplet-associated factors, including patatin-like phospholipase domain-containing 3 (PNPLA3), transmembrane 6 superfamily member 2 (TM6SF2), and 17β-hydroxysteroid dehydrogenase (HSD17B) 11 and 13, with the aim of proposing them, and consequently lipid droplets, as new potential therapeutic options [9]. Instead, regarding IPMN, other authors described new factors that could help to diagnose this type of benign neo-formation in order to avoid its evolution into a malignant phenotype. In particular, they structured their work by dividing low- and high-risk factors and identified some potential biomarkers, including some involved in metabolic pathways, that can help to identify IPMNs that have a high risk to become cancerous [10].

In conclusion, new important evidence emerged from this Special Issue, leading us to further encourage the study of the metabolism in PDAC by taking into consideration alterations of specific metabolic proteins and mitochondrial arrangement/function to develop new therapeutic strategies that could improve the survival rate of PDAC patients.
